# Thanks to all those who reviewed for *Trials* in 2014

**DOI:** 10.1186/s13063-015-0571-y

**Published:** 2015-02-22

**Authors:** Douglas G Altman, Curt D Furberg, Jeremy M Grimshaw

**Affiliations:** Centre for Statistics in Medicine, University of Oxford, Botnar Research Centre, Windmill Road, Oxford, OX3 7LD UK; Division of Public Health Sciences, Wake Forest University School of Medicine, Medical Center Boulevard, Winston-Salem, NC 27157-1063 USA; Clinical Epidemiology Programme, Ottawa Hospital Research Institute and Department of Medicine, University of Ottawa, 501 Smyth Road, Ottawa, ON K1H 8 L6 Canada

## Abstract

A peer-reviewed journal would not survive without the generous time and insightful comments of the reviewers, whose efforts often go unrecognized. Although final decisions are always editorial, they are greatly facilitated by the deeper technical knowledge, scientific insights, understanding of social consequences, and passion that reviewers bring to our deliberations. For these reasons, the Editors-in-Chief and staff of the journal warmly thank the 610 reviewers whose comments helped to shape *Trials*, for their invaluable assistance with review of manuscripts for the journal in Volume 15 (2014).

**Purva Abhyankar**

UK

**Hatem Abu Hashim**

Egypt

**Clive E Adams**

UK

**Joy Adamson**

UK

**Christopher Adlbrecht**

Austria

**Luciano Agati**

Italy

**Rakesh Aggarwal**

India

**Mahboob Alam**

USA

**Eric Albrecht**

Switzerland

**Muktar Aliyu**

USA

**Kelli Allen**

USA

**Osvaldo Almeida**

Australia

**Constantine Amditis**

Australia

**Gavin Andrews**

Bahamas

**Djillali Annane**

France

**Massimo Antonelli**

Italy

**Ane Appelt**

Denmark

**Bruce Arroll**

New Zealand

**Deborah Ashby**

UK

**Lisa Askie**

Australia

**George Atkinson**

USA

**Peter Bacchetti**

USA

**Franz G. Bader**

Germany

**Gillian Baer**

UK

**Anne-Marie Bagnall**

UK

**Sean Bagshaw**

Canada

**Patricia Ballesta**

Spain

**Karla Ballman**

USA

**Barbara Bardoni**

France

**Neil Barnes**

UK

**Guido Bastiaens**

Netherlands

**Erika Baum**

Germany

**Shrujal Baxi**

USA

**David Beard**

UK

**Beatrice Beck-Schimmer**

Switzerland

**Vincenzo Berghella**

USA

**Julie Bernhardt**

Australia

**Pietro Bertini**

Italy

**Martin Bertrand**

France

**Mireia Besalú**

Spain

**Zhaoxiang Bian**

Hong Kong

**José Bilbao**

Spain

**Ariella Binik**

UK

**Yvonne Birks**

UK

**Jonathan Bisson**

UK

**Steven Blair**

USA

**Jane Blazeby**

UK

**Ashley Bonner**

Canada

**Jonathan Boote**

UK

**Ron Borland**

Australia

**Mark Borthwick**

UK

**Josée Bouchard**

Canada

**Peter Bower**

UK

**Mike Bradburn**

UK

**Patrick Brady**

USA

**Michael Brainin**

Austria

**Thijs Brandsma**

Netherlands

**Christopher Bray**

UK

**Daniel Bressington**

Hong Kong

**Laurent Brochard**

Canada

**Marianne Brodmann**

Austria

**Gert Bronfort**

USA

**Richard Brown**

USA

**Sarah Brown**

UK

**Sandra Bucci**

UK

**Stefan Budde**

Germany

**Lorraine Buis**

USA

**Bryan Burford**

UK

**Tom Burns**

UK

**Martin Burtscher**

Austria

**Phyllis Butow**

Australia

**Marc Buyse**

Belgium

**Agnès Caille**

France

**Michael Campbell**

UK

**Bridget Candy**

UK

**Daniel Carr**

USA

**Christopher Carroll**

UK

**Maija Castren**

Finland

**Cheejen Chang**

Taiwan

**Xiaorong Chang**

China

**Sue Channon**

UK

**Baoying Chen**

China

**Wei Chen**

USA

**Yiyi Chen**

USA

**Christopher Chen**

Singapore

**Dongquan Chen**

USA

**Ji Cheng**

Canada

**Wai Tong Chien**

Hong Kong

**Pietro Chiurazzi**

Italy

**Sun-Mi Choi**

Korea, South

**Babak Choodari-Oskooei**

UK

**Chun-Hung Chu**

Hong Kong

**Rong Chu**

Canada

**Roberto Cirocchi**

Italy

**David Clarke**

UK

**Jan Clarkson**

UK

**Elisabeth Coart**

Belgium

**Erik Cobo**

Spain

**Christopher Coffey**

USA

**Dena Cohen**

UK

**Judith Cohen**

UK

**Natasha Cohen**

Canada

**Ben Colagiuri**

Australia

**Tim Colbourn**

UK

**Dave Collett**

UK

**Deborah Joanne Cook**

Canada

**Cindy Cooper**

UK

**Simon Coulton**

UK

**Peter Alan Coventry**

UK

**Peter Craig**

UK

**Holger Cramer**

Germany

**Georgina Crichton**

Australia

**Christine Cronin**

USA

**William Crown**

USA

**Allan Cyna**

Australia

**Sara Czaja**

USA

**Dave Dagnan**

USA

**Rafael Dal-Ré**

Spain

**Sarah Damery**

UK

**Antoni Davalos**

Spain

**Colin Dayan**

UK

**Sabina De Geest**

Switzerland

**Harry J. De Koning**

Netherlands

**Jorge De La Torre**

USA

**Daniele De Luca**

Italy

**Christian De Mey**

Germany

**Jon Deeks**

UK

**Emilia D'elia**

Italy

**Ingrid Demmelmaier**

Sweden

**Catherine D'este**

Australia

**Niveditha Devasenapathy**

India

**Francesco Di Mola**

Italy

**Sean Dinneen**

Ireland

**Beate Ditzen**

Germany

**Dennis Dixon**

USA

**Mary Dixon-Woods**

UK

**Peter Dodek**

Canada

**Gordon Doig**

Australia

**Tara Donker**

Australia

**Allan Donner**

Canada

**Jenny L. Donovan**

UK

**Peter Doshi**

USA

**George Dowswell**

UK

**Rohan D'souza**

Canada

**Joel Dubin**

Canada

**Chantale Dumoulin**

Canada

**Graham Dunn**

UK

**David Daniel Ebert**

Germany

**Allison Ross Eckard**

USA

**Diana Elbourne**

UK

**C Kristian Enestvedt**

USA

**Steven Engebretson**

USA

**Tracy Epton**

UK

**Mert Erkan**

Germany

**Tom Fahey**

Ireland

**Muhammad Faisal**

Pakistan

**Anna Falanga**

Italy

**Morag Farquhar**

UK

**Paul Farrand**

UK

**Michael Fehlings**

Canada

**Dean Fergusson**

Canada

**Ricardo Fernandes**

Portugal

**Frederick Ferris**

USA

**Shona Fielding**

UK

**Graziella Filippini**

Italy

**Abe Fingerhut**

France

**Signe Flottorp**

Norway

**Maelán Fontes Villalba**

Spain

**Andrew Hugh Ford**

Australia

**Christen Fornadel**

USA

**Laura Forsythe**

USA

**Chris Foy**

UK

**Marlene Fransen**

Australia

**Tobias Freund**

Germany

**Lawrence Friedman**

USA

**Xavier Fritel**

France

**Ivana Furimsky**

Canada

**Carrol Gamble**

UK

**Simon Gates**

UK

**Johnson George**

Australia

**Jean-Pierre Gerard**

France

**Prithwijit Ghosh**

USA

**Mark Gillies**

Australia

**Montserrat Girabent**

Spain

**Timothy Girard**

USA

**Lise Gluud**

Denmark

**Charlie Goldsmith**

Canada

**Manuel Gomes**

UK

**Primitivo Gómez Cardero**

Spain

**Mithat Gonen**

USA

**John Gonzalez**

UK

**Marjorie Good**

USA

**Adam Goode**

USA

**Jayashree Gopal**

India

**Jonathan Graffy**

UK

**Sean Grant**

UK

**Jocelyn Gravel**

Canada

**Walter Gregory**

UK

**Andrew Grey**

New Zealand

**Andrew Grieve**

UK

**James Griffin**

UK

**Xavier Griffin**

UK

**Ulla Griffiths**

UK

**Freedom Nkhululeko Gumedze**

South Africa

**Olivier Guyen**

Switzerland

**Ok Kyung Ham**

Korea, South

**James Hanley**

Canada

**Paul Harden**

UK

**Nicola Harman**

UK

**Sue Harnan**

UK

**Robert Hatala**

Slovakia

**Charles Heilig**

USA

**Asgeir R. Helgason**

Sweden

**Paul Hendrick**

UK

**Matthias Hermann**

Switzerland

**Sharlene Hesse-Biber**

USA

**Sarah Hetrick**

Australia

**Christo Heunis**

South Africa

**Michelle Heys**

UK

**Daniel Hind**

UK

**Christoph Hofer**

Switzerland

**Marie Hogan**

USA

**Maxi Holland**

Germany

**Nina Homayoon**

Austria

**Richard Hooper**

UK

**Val Hopwood**

UK

**Dell Horey**

Australia

**Kristina Dupont Hougaard**

Denmark

**Louise Michele Howard**

UK

**Michael Howley**

USA

**Jui-Chien Hsieh**

Taiwan

**Bo Hu**

USA

**Dominique Hubert**

France

**John Hummel**

USA

**Scott Hummel**

USA

**David Hunter**

Australia

**Myra Hunter**

UK

**John Hustad**

USA

**Peter Hutchinson**

UK

**Ana Iltis**

USA

**Joanne Ingwall**

USA

**George Ioannidis**

Canada

**Sameena Iqbal**

Canada

**Khalida Ismail**

UK

**Kristina Jackson**

USA

**Adam Jacobs**

UK

**George Jallo**

USA

**Lewis James**

UK

**Nicholas James**

UK

**Murali Janakiram**

USA

**Thomas Janssens**

Belgium

**Pawel Jastreboff**

USA

**Martin Jenkins**

UK

**Gail Jensen**

USA

**Kristin Jerger**

USA

**Honghua Jiang**

USA

**Antony Johansen**

UK

**Robert Johansson**

Sweden

**Kent Johnson**

Australia

**Ashley Jones**

UK

**David Jones**

UK

**Patricia Jones**

USA

**Sue Jordan**

UK

**Lawrence Joseph**

Canada

**Andrew Judge**

UK

**Steven Julious**

UK

**Michael Kaess**

Germany

**Juliana Kain**

Chile

**Norma Kanarek**

USA

**Steve Karas**

USA

**Sunil Kari**

India

**Manuella Kaster**

Brazil

**Thomas Kaulhausen**

Germany

**Walter Kernan**

USA

**Sally Kerry**

UK

**Line Kessel**

Denmark

**David Kessler**

UK

**Arndt Kiessling**

Germany

**Nanteza Gladys Kigozi**

South Africa

**Seung-Nam Kim**

USA

**Soo Wan Kim**

Korea, South

**Peter Kimani**

UK

**Jonathan Kimmelman**

Canada

**Ann Louise Kinmonth**

UK

**Kevin Kissling**

USA

**Heather Kitzman-Ulrich**

USA

**Lawrence Kleinman**

USA

**Roman Kloeckner**

Germany

**Sarah Knowles**

UK

**Kristen Knutson**

USA

**Seok-Jae Ko**

Korea, South

**Yun Hyung Koog**

Korea, South

**Riikka Korja**

Finland

**Belchin Kostov**

Spain

**Helena Kraemer**

USA

**Michael Krams**

USA

**Jayashri Kulkarni**

Australia

**Lindsay Kuroki**

USA

**Sunoh Kwon**

USA

**Jan Kyselovic**

Slovakia

**Christopher Labos**

Canada

**Jacques Lacroix**

Canada

**Trudie Lang**

UK

**Marilyne Lange**

Netherlands

**Helena Länsimies-Antikainen**

Finland

**Marissa Lassere**

Australia

**Dimitrios Lathyris**

Greece

**Eric Lau**

Hong Kong

**Romy Lauche**

Germany

**Alexandre Lautrette**

France

**Julia Lawton**

UK

**Gerald Lebovic**

Canada

**Myeong Soo Lee**

Korea, South

**Ellen Lee**

UK

**Seungmin Lee**

Korea, South

**Stanley Lemeshow**

USA

**Isabel Leroux-Roels**

Belgium

**Stefan Leucht**

Germany

**Daniel Levin**

USA

**Beth Lewis**

USA

**Ruth Lewis**

UK

**Joel Lexchin**

Canada

**Ai-Hsien Li**

Canada

**Ai-Hsien Li**

Taiwan

**Shaojung Li**

Taiwan

**Min Li**

Hong Kong

**Fan-Rong Liang**

China

**Lalita Limaye**

India

**Piotr Lipiec**

Poland

**Jianping Liu**

China

**Cun-Zhi Liu**

China

**Teresa Liu-Ambrose**

Canada

**Xiong Lize**

China

**Francesco Locatelli**

Italy

**S. Scott Lollis**

USA

**Carl Lombard**

South Africa

**J Longhurst**

USA

**Colleen Loo**

Australia

**Purificación Lopez**

Spain

**Travis Loux**

USA

**Aiping Lu**

Hong Kong

**Jon Lurie**

USA

**David Lussier**

Canada

**Jinhui Ma**

Canada

**Andrew Maas**

Belgium

**Lynette MacKenzie**

Australia

**Graeme MacLennan**

UK

**Hugh MacPherson**

UK

**Michael Tvilling Madsen**

Denmark

**Albane B.R. Maggio**

Switzerland

**Tanya Mailhot**

Canada

**Valerio Mais**

Italy

**Laxmaiah Manchikanti**

USA

**Jun Mao**

USA

**Maura Marcucci**

Canada

**Neil Marlow**

UK

**Tony Marson**

UK

**Arturo MartIacute-Carvajal**

Venezuela

**David Martin**

Australia

**Monica Martinez-Cengotitabengoa**

Spain

**Julia Mascherbauer**

Austria

**Karen Maschke**

USA

**Nancy Mayo**

Canada

**Lawrence Mbuagbaw**

Cameroon

**Amanda Mcintyre**

Canada

**Sharon Mckinley**

Australia

**Roberto Méndez-Sánchez**

Spain

**Hanna Merk**

Sweden

**Sally Merry**

New Zealand

**Luc Mertens**

Canada

**Luca Miceli**

Italy

**Christoph Michalski**

Germany

**Oliver Micke**

Germany

**Sandy Middleton**

Australia

**Jordan Miller**

Canada

**Vanessa Miller**

USA

**Jesse Mills**

USA

**Nicola Mills**

UK

**Erich Minar**

Austria

**Ben Mol**

Afghanistan

**Alan Montgomery**

UK

**Hayley Moore**

UK

**Eva Morris**

UK

**Tim Morris**

UK

**Joanna Morrison**

Nepal

**William Mortenson**

Canada

**Yoshiharu Motoo**

Japan

**Lawrence Moulton**

USA

**Brendan Mulhern**

UK

**Caroline Murphy**

UK

**Gordon Murray**

UK

**Jonah Musa**

Nigeria

**Shannon Myers Virtue**

USA

**Luigi Naldi**

Italy

**Melissa Napolitano**

USA

**Silvana Naredi**

Sweden

**Thais Nascimento**

Canada

**Efrat Neter**

Israel

**Marie-Louise Newell**

UK

**Tim Newton**

UK

**Paul Nietert**

USA

**Teemu Niiranen**

Finland

**Stavros Nikolakopoulos**

Netherlands

**Magnus Nilsson**

Sweden

**Curtis Noonan**

USA

**Tine Nordgreen**

Norway

**John Norrie**

UK

**Paulina Nowicka**

Sweden

**Xavier Nuñez**

Spain

**Stacy O'blenes**

Canada

**Alicia O'cathain**

UK

**Eraldo Occhetta**

Italy

**Lang'o Odondi**

UK

**Adefowope Odueyungbo**

USA

**Michael Okun**

USA

**Goran Olivecrona**

Sweden

**Stefano Omboni**

Italy

**Robert O'neill**

USA

**Anthony Ormerod**

UK

**Matthew Page**

Australia

**Christopher Parshuram**

Canada

**Nick Parsons**

UK

**Megan Passey**

Australia

**James Paul**

Canada

**Janet Peacock**

UK

**Nuria Perez-Alvarez**

Spain

**Leigh Perreault**

USA

**Sean Perrin**

UK

**Paolo Persichetti**

Italy

**Christopher Phillips**

USA

**Sean Philpott-Jones**

USA

**Lira Pi**

USA

**Claude Pichonnaz**

Switzerland

**Davide Pietropaoli**

Italy

**Stefan Pilz**

Austria

**Carlos E. Pinfildi**

Brazil

**Paul Pinsky**

USA

**Daniel Pinto**

Portugal

**Robert Platt**

Canada

**Jenny Ploeg**

Canada

**Gregory Pond**

Canada

**Rudolf Poolman**

Netherlands

**Raphael Porcher**

France

**Martin Posch**

Austria

**John Powell**

UK

**Lindsay Prior**

UK

**Riccardo Proietti**

Italy

**Audrey Prost**

UK

**Eleanor Pullenayegum**

Canada

**Daryl Pullman**

Canada

**Helena Quesada**

Spain

**Antoine Rabbat**

France

**James Raftery**

UK

**Jochen Raimann**

USA

**Inderpal Randhawa**

USA

**Nicolas Rasmussen**

Australia

**Stefania Redolfi**

France

**Sunita Rehal**

UK

**Clare Relton**

UK

**Matthieu Resche-Rigon**

France

**Juliette Richetin**

Italy

**Dan Riddle**

USA

**Rachel Riechelmann**

Brazil

**Robert Rintoul**

UK

**Maria Rosaria Rizzo**

Italy

**Wendy Robertson**

UK

**Reitze Rodseth**

South Africa

**Rhonda Rosychuk**

USA

**Àngel Ruiz**

Spain

**Milind Ruke**

India

**Roman Rukwied**

Germany

**Clare Rutterford**

UK

**Everardo Saad**

Brazil

**Tsuneaki Sadanaga**

Japan

**Brodie Sakakibara**

Canada

**Jorge Salluh**

Brazil

**Zainab Samaan**

Canada

**John Sampalis**

Canada

**Peter Sandercock**

UK

**Carlos Santos-Gallego**

USA

**David Saunders**

Thailand

**Mary Sawtell**

UK

**Simonetta Scalvini**

Italy

**Dirk Schadler**

Germany

**Stephen Schaffer**

USA

**Bawarjan Schatlo**

Germany

**Roberta Scherer**

USA

**Ulrike Schmidt**

UK

**Simon Schneider**

Germany

**Rosa Schnyer**

USA

**David Schriger**

USA

**Wilhelm Schulte-Mattler**

Germany

**Kenneth Schulz**

USA

**Julio José Secades Ruiz**

Spain

**Sukhdev Sembi**

UK

**Mehmet Sen**

UK

**Kok-Min Seow**

Taiwan

**Andreas Serra**

Switzerland

**Dean A. Sewell**

UK

**Fabrizio Sgolastra**

Italy

**Dr Shalimar**

India

**Hongcai Shang**

China

**Surya Kant Sharma**

India

**Anjail Sharrief**

USA

**Byung-Cheul Shin**

Korea, South

**Joseph Patrick Shott**

USA

**Ian Shrier**

Canada

**Muhammed Siddiqui**

UK

**Julius Sim**

UK

**Fiona Simpson**

Australia

**Sergio Siragusa**

Italy

**Lene Theil Skovgaard**

Denmark

**Susan Slaughter**

Canada

**Martin Smalbrugge**

Netherlands

**Alexey Smetkin**

Russian Federation

**Helen Snooks**

UK

**Claire Snowdon**

UK

**Priscilla Soares**

Brazil

**Mikael Sodergren**

UK

**Masoud Solaymani-Dodaran**

Iran

**Daniel Solomon**

USA

**Kjetil Soreide**

Norway

**Harald Sourij**

UK

**Sonia Souto**

Spain

**Anshu Srivastava**

India

**Cesare Stabilini**

Italy

**Amanda Staiano**

USA

**Nigel Stallard**

UK

**Simon Stanworth**

UK

**Emily Stockings**

Australia

**Magdalena Strach**

Poland

**Bela Suki**

USA

**Frank Sullivan**

UK

**Frank Sullivan**

Canada

**Qi Sun**

China

**Chris Sutton**

UK

**Nao Suzuki**

Japan

**Vigdis Sveinsdottir**

Norway

**Matthew Robert Sydes**

UK

**Yutaka Takeda**

Japan

**Monica Taljaard**

Canada

**Navdeep Tangri**

Canada

**David Tappin**

UK

**Warwick Teague**

Australia

**Melissa Teo**

Singapore

**Alexandre Terrier**

Switzerland

**Ekmel Tezel**

Turkey

**George Thanassoulis**

Canada

**Achilleas Thoma**

Canada

**Brett Thombs**

Canada

**Merran Toerien**

UK

**Mattie Tops**

Netherlands

**Shaun Treweek**

UK

**Janette Turner**

UK

**Sheila Turner**

UK

**Laura Twyman**

Australia

**Christi Ulmer**

USA

**Thongchai Vachirarojpisan**

Thailand

**Luc Valiquette**

Canada

**Tjeerd Van Staa**

UK

**Hilde Verbeek**

Netherlands

**Johan Verbraecken**

Belgium

**Emily Vertosick**

USA

**Roland Vetter**

Germany

**Miguel Vicco**

Argentina

**Andrew Vickers**

USA

**Marcos Vidal Melo**

USA

**Marta Vilaró**

Spain

**Sophocles Voineskos**

Canada

**Julia Wade**

UK

**Adrian Wagg**

Canada

**Rolf Wahlstrom**

Sweden

**Stephen John Walters**

UK

**Shu Wang**

China

**Germain Weber**

Austria

**Angela Webster**

Australia

**Jacqui Webster**

Australia

**Martin O. Weickert**

UK

**Birgit Weinberger**

Austria

**Gesine Weser**

Germany

**Helen Whitford**

Australia

**Natasha Wiebe**

Canada

**Alishia Williams**

Australia

**Michelle Wise**

New Zealand

**Claudia Witt**

Switzerland

**Claudia Witt**

Germany

**Barbara Wizner**

Poland

**Johannes Woitzik**

Germany

**Anna Wolters**

Netherlands

**Trecia Wouldes**

New Zealand

**Darong Wu**

China

**Justin Wu**

Hong Kong

**Yu-Tai Wu**

USA

**Taixiang Wu**

China

**Raphael Wurm**

Austria

**Rudolf P. Wüthrich**

Switzerland

**Jielai Xia**

China

**Lize Xiong**

China

**Bin Xu**

China

**Chenglin Ye**

Canada

**Chiu Yen Ling**

Taiwan

**Wing-Fai Yeung**

Hong Kong

**Fang Yu**

USA

**Zhou Yujie**

China

**Muhamad Saiful Bahri Yusoff**

Malaysia

**Bai-Yun Zeng**

UK

**Qinhong Zhang**

China

**Lihui Zhao**

USA

**Linda Zhong**

Hong Kong

**Bingmei Zhu**

China

**Merrick Zwarenstein**

Canada

